# Remote Symptom Assessment in Palliative Care: Patient and Clinician Perspectives

**DOI:** 10.1093/geroni/igaf122.1794

**Published:** 2025-12-31

**Authors:** Irina Mindlis, Emily Bivona-Maldonado, Maureen Ekwebelem, Catherine Samaniego, Samprit Banerjee, Zilong Yu, Daniel Shalev

**Affiliations:** Rutgers University, New York, New York, United States; Weill Cornell Medicine, New York, New York, United States; Weill Cornell Medicine, New York, New York, United States; Weill Cornell Medicine, New York, New York, United States; Weill Cornell Medicine, New York, New York, United States; Weill Cornell Medicine, New York, New York, United States; Weill Cornell Medicine, New York, New York, United States

## Abstract

Palliative care (PC) aims to improve quality of life by managing physical and psychological symptoms. High symptom burden is a primary driver of PC referral, yet gaps in symptom assessment limit the impact of PC interventions. Given that ambulatory PC visits often occur weeks apart, day-to-day symptom patterns may go unrecognized and recall bias during appointments may further hinder the quality of data on which treatment decisions are based. Remote symptom assessment (RSA), the collection of symptom data outside of clinical visits via digital or telephonic methods, offers a potential solution but remains underutilized. We aimed to understand the perspectives of PC patients and clinicians on RSA to improve symptom monitoring in ambulatory PC. We conducted a descriptive-exploratory qualitative study using semi-structured interviews with 10 PC patients and 9 PC clinicians at a single ambulatory PC program. Thematic analysis, using hybrid inductive-deductive coding, identified key themes regarding RSA’s benefits, desired features, and challenges. Participants viewed RSA as a tool to empower both patients and clinicians in managing symptoms. Desired features included facile user interfaces, structured reminder systems, and alerts for clinical changes. Participant concerns included technical complexity, time burden, and information burden. Ultimately, both patients and clinicians felt that RSA could enhance symptom management in the ambulatory PC setting, but only if developed with high usability and minimized burden. These findings highlight the need for user-centered RSA tools tailored to the ambulatory PC setting. Future research should focus on co-designing and piloting RSA platforms to optimize feasibility and clinical integration.

